# Specimen Size Effect on the Tensile Properties of Rolled Steel of Long-Term-Operated Portal Crane

**DOI:** 10.3390/ma16083017

**Published:** 2023-04-11

**Authors:** Olha Zvirko, Ihor Dzioba, Myroslava Hredil, Robert Pała, Oleksandr Oliynyk, Piotr Furmańczyk

**Affiliations:** 1 Department of Diagnostics of Materials Corrosion-Hydrogen Degradation, Karpenko Physico-Mechanical Institute of the NAS of Ukraine, 5 Naukova St., 79060 Lviv, Ukraine; 2 Department of Machine Design, Faculty of Mechatronics and Mechanical Engineering, Kielce University of Technology, Av. 1000-an. of Polish State, 7, 25-314 Kielce, Poland; 3 Department of Lifting and Transport Machines and Engineering of Port Technological Equipment, Odessa National Maritime University, 34, Mechnikova St., 65029 Odesa, Ukraine

**Keywords:** steel, anisotropy, long-term operation, tensile properties, elongation, thickness influence, impact toughness

## Abstract

This paper presents the research results on the mechanical behavior of the low-carbon rolled steel of a sea portal crane after a 33-year operation depending on the operational stresses and rolling direction in order to assess its serviceability. The tensile properties of steels were investigated using rectangular cross-section specimens with different thicknesses and the same width. Strength indicators were slightly dependent on the considered factors (operational conditions, the cutting direction, and thickness of specimens). However, a clear trend of higher ultimate strength for thinner specimens was noticed, especially in the case of more brittle material due to its operational degradation. Plasticity of the tested steel specimens was more sensitive to the influence of the above-mentioned factors than strength but less sensitive than impact toughness. Uniform elongation was slightly less for thinner specimens regardless of the investigated steel state or the orientation of specimens relative to the rolling direction. The post-necking elongation was lower for transversal specimens compared with longitudinal ones, and the effect was more significant when testing steel with the lowest brittle fracture resistance. Among the tensile properties, non-uniform elongation was demonstrated to be the most effective for assessing the operational changes in the state of rolled steels.

## 1. Introduction

Stress-strain curves are widely used to determine tensile mechanical properties of materials, namely, strength and plasticity. Together with fracture toughness at quasi-static loading conditions or impact toughness, these characteristics are considered to be the basic mechanical properties of the material, and they are most often included in regulations for structural steels. The mechanical properties of steels are dependent not only on intrinsic microstructural features, such as the grain size and structure of the precipitated particles [1,2,3,4], but also on extrinsic testing conditions, such as strain rate [5,6], specimen geometry and size [4,7,8,9,10,11,12,13], and temperature [14,15,16]. Therefore, testing conditions and the geometry and dimensions of specimens are regulated by standards [17,18]. However, tensile specimens with different thicknesses and geometries are sometimes used [1,9], especially for testing materials with geometric size limitations, such as a metal sheet. Moreover, there is no exact requirement for the specimen thickness in standards.

Specimen-size effect on the tensile properties has been considerably studied recently for materials with different microstructures. In many cases, specimens with a rectangular cross section were used, and their size influenced the tensile properties of materials [1,7,9,19,20,21], including strength and plasticity. The influence of the gauge length on the tensile properties was studied in [7], showing that both total elongation and uniform elongation decrease with an increase in the gauge length/width ratio. At the same time, it was demonstrated that the intrinsic deformation is not dependent on the gauge length [7]. It was revealed in [9] that upper and lower yield strength, tensile strength, and post-necking elongation increase with increasing thickness of specimens with a rectangular cross section. Both uniform elongation and post-necking elongation increase with increasing specimen thickness, and such changes are mainly attributed to the necking behavior of the specimens [7].

Anisotropy of materials is a commonly observed phenomenon, especially for steels produced by rolling. Therefore, the mechanical properties of rolled steels vary depending on the direction along which they are measured [6,9,22,23,24,25,26,27,28]. The main causes of anisotropy are predominant crystallographic orientation (texture) and alignment and distribution of secondary phases, such as inclusions, in the microstructure. A commonly accepted experimental test used to characterize anisotropy of properties of textured rolled steels is the uniaxial tensile test using specimens cut out in different orientation relative to the rolling direction. Three main orientations are most often considered, namely, longitudinal (along the rolling of the plate), transverse (transverse to the rolling direction of the plate), and short transverse (perpendicular to the rolled plate surface). Nevertheless, the regulating documents and standards do not always specify the direction of specimen cutting relative to rolling, while longitudinal specimens are usually used because transversal or short transversal ones are sometimes impossible to cut out due to the size limitations of an object. One example of this is the case of testing thin-wall pipes with a small diameter made of rolled sheet steels [22].

In general, the resistance to fracture is the lowest for short transversal specimens and the highest for longitudinal ones [25,27]. It is usually the result of a weaker cohesion between the layers of texture and between the matrix and non-metallic inclusions elongated along the rolling direction, which promotes damage evolution in those sites [25,26,27,29]. In transversal specimens, the fracture propagates along the rolling direction, and it is facilitated due to existing areas with a reduced cohesive strength potentially susceptible to delamination or damages in the form of delaminations in the case of long-term-operated steel [26,27]. As a result, fracture mode is more brittle for transversal specimens compared with longitudinal ones [27,30].

In addition, operational degradation of structural steels [31,32,33,34,35,36,37,38,39,40] should also be taken into account. It is well-known that the long-term operation of steels often leads to a significant loss of the mechanical properties that ensure the serviceability of the material under certain loading conditions. The main peculiarity of in-service degradation consists primarily in the development of in-bulk damages at the nano- and micro-scale [26,41] that lead to a drop in plasticity and brittle fracture resistance and often cause abrupt failures. A crucial role of operational cyclic loading in in-bulk degradation of carbon steels of seaport hoisting and transporting equipment has been recently demonstrated in research [38,39]. Structures operated long-term under cyclic loading are subjected to strain hardening as well as fatigue crack initiation. Steel degradation manifests itself in a significant deterioration of impact toughness, and the higher the stress level, the lower the resistance to brittle fracture. For the rolled steels, cohesive strength between the matrix and non-metallic inclusions, elongated in the rolling direction, significantly reduces during operation [35,37]. It leads to enhancing the anisotropy of plasticity and the resistance to brittle fracture. The influence of long-term operation on the anisotropy of rolled steels is still under consideration. Moreover, studies [27,31] have shown that the anisotropic mechanical behavior of rolled pipeline steels is influenced by long-term operation. Recent studies [31,32,37,42] have demonstrated that transverse specimens were more suitable for the assessment of the degradation degree of steels. Therefore, an important research issue is the effect of damages in rolled steel due to operational degradation on the size factor manifestation based on the stress-strain curves.

The objective of this paper is to evaluate the mechanical behavior of portal crane rolled steel depending on operational stresses and rolling direction in order to assess its serviceability.

## 2. Materials and Testing Methods

**Figure 1:**
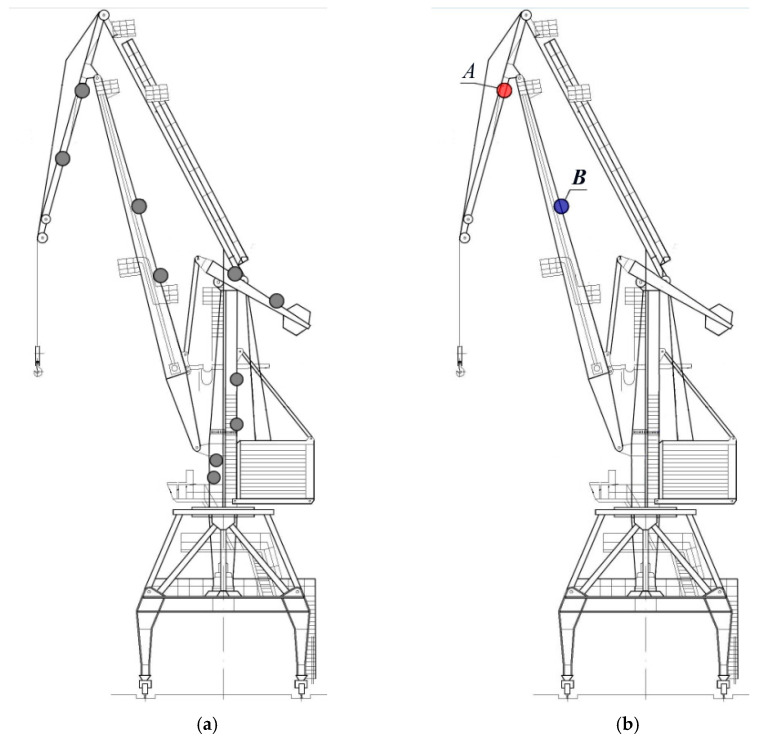
Schemes of a portal crane indicating the points for cyclic stress evaluation (**a**) and units chosen for present research (**b**): *A*–steel A, *B*–steel B.

The object of research is a portal crane (Figure 1) made of rolled sheet steel (low-carbon steel with ferrite-pearlite structure) after a 33-year operation in a seaport in Ukraine [39]. The averaged chemical composition of the steel was as follows: mass. %: 0.17 C; 0.23 Si; 0.54 Mn; 0.11 Cr; 0.01 S; 0.01 P; 0.10 Cu; and Fe—balance. The crane was manufactured from the same grade steel with the basic mechanical properties that met the specifications they were supplied to. The crane was subjected to approximately 5.3 × 10^6^ cycles during operation.

The crane was operated under the action of cyclic loading and environmental impact. Operational factors which affect the serviceability of seaport hoisting and transporting equipment are considered in detail in [39]. Cyclic stresses are generally supposed to be the main factor in the operational degradation of portal crane metal [33,34,38,39]. Various crane units (Figure 1a) were subjected to mechanical cyclic loads of different intensities, which could contribute to operational degradation of the metal of certain structural elements due to strain hardening as a main mechanism of degradation. Combining the strain gauge method and calculations, cyclic stress ranges at the sheet surface Δσ_e_ have been evaluated for certain conditions of a loading-unloading cycle of the crane close to operational ones. First of all, the calculation of the stress-strain state of different sections of a portal crane was carried out using the finite element method in the ANSYS. After that, the most critical section zones and, accordingly, the sites for adjustment of the strain gauges on the portal crane were determined, and then strain measurements were carried out. Strains at different points of the crane (ten in total, indicated in Figure 1a) were measured using strain gauges under the loading-unloading cycle with an applied load close to the operational one. After that, cyclic stress ranges at the sheet surface were determined as the difference in stresses measured at the maximum cyclic load (crane under loading) and in unloading conditions. The applied technique is described in detail in [38]. It should be noted that the results of the numerical evaluation of stress distributions in crane units and field measurements practically coincided.

**Figure 2:**
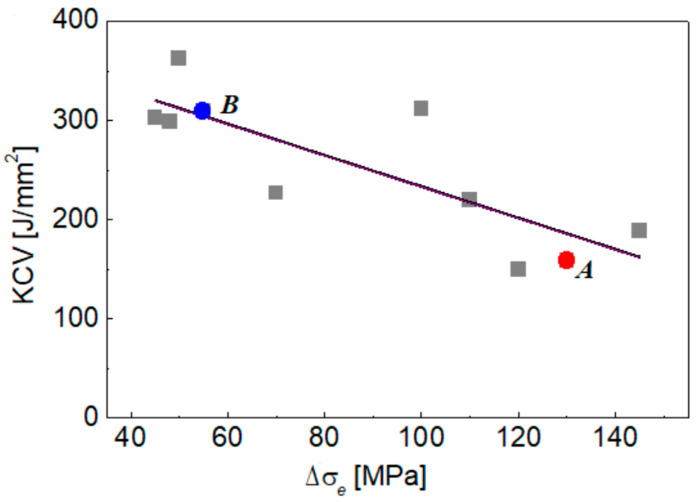
Dependence between impact toughness KCV and cyclic stress range Δσ_e_ for steel cut out from various crane units. Units chosen for present research (A and B) are marked.

Considering previously evaluated impact toughness for the metal of the tested crane units [38], a general regularity has been found for all tested samples (Figure 2): the more cyclic stress range, the less impact toughness (e.g., brittle fracture resistance). It was supposed that the metal of the tested sections would not be identical in the initial state, and that their mechanical properties might slightly vary from piece to piece. In addition, the thickness of the plates also varied (10; 12; 16 and 25 mm). However, given the revealed regularity, it is suggested that operational conditions (cyclic stresses) are the crucial factor causing an enormously low-impact toughness of the most stressed components of the operated crane.

Based on the determined cyclic stress ranges, two crane sections with high and low cyclic stress ranges were chosen for further mechanical testing, as illustrated in Figure 1b. The first one is a lower shelf of the jib made of steel sheet with a thickness of 16 mm (steel A), and the second is a back shelf of the boom with a sheet thickness is 12 mm (steel B). The difference in Δσ_e_ values is significant for the tested crane sections: 55.0 MPa and 130.0 MPa for steels A and B, respectively (Table 1). Therefore, it can be suggested that the degradation degree of the studied steels is different. Although the degradation degree of steel from each section can’t be quantitatively evaluated because of a lack of information about its initial characteristics, the present research is believed to provide at least a qualitative comparison of the actual technical state of two crane units subjected to significantly different operational loads under long-term (33 years) operation.

**Figure 3:**
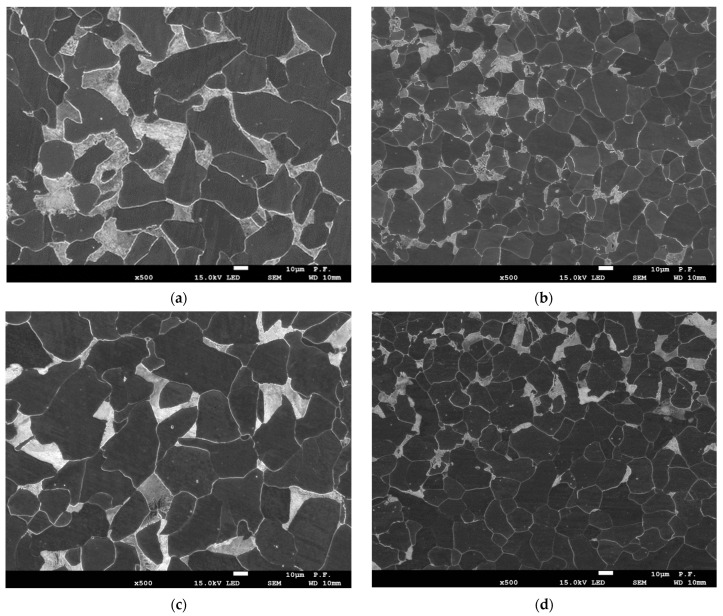
The SEM photographs of steel microstructure in longitudinal (**a**,**b**) and transverse directions (**c**,**d**): (**a**,**c**)—steel A; (**b**,**d**)—steel B.

The microstructures of the steels were observed using scanning electron microscopy (SEM). The studied steels had similar ferrite-pearlite microstructures, as shown in Figure 3. The microstructure of steel B (Figure 3b,d) was characterized by smaller average grain size compared to that of steel A (Figure 3a,c).

**Test methods**. We tested the steel from two crane sections, A and B (Figure 1), which differ significantly by the determined cyclic stress ranges (Table 1).

Since the degradation of metal manifests itself firstly in the drop of brittle fracture resistance due to the realization of a less energy-consuming fracture mechanism [33,35,37,38,39], impact toughness as a measure of the resistance to brittle fracture has been evaluated for the metal from both tested crane sections. Standard specimens 10 mm × 10 mm × 55 mm with a V-type notch for impact toughness testing were cut out from crane sections in longitudinal (L-T, direction of the global crack propagation was transverse) and transverse (T-L, the crack was propagated in longitudinal direction) directions relative to the plate processing geometry. Tests were performed according to ASTM E23-07a standard [43].

Basic mechanical properties were evaluated by tensile testing of flat specimens cut out longitudinally and transversely to the rolling direction of steel sheets. Two series of specimens with different gauge dimensions (thickness t × width × length) were used: 5.0 × 3.5 × 20.0 mm and 1.2 × 3.5 × 20.0 mm. Thickness is considered crucial in specimen distinctions; manufacturing flat specimens of a small thickness is easier than cylindrical ones. The research plan thus included eight types of specimens used for experiments (Table 2). Specimens were subjected to uniaxial tensile tests with the strain rate 3∙10^−3^ s^−1^ in air at ambient temperature according to ASTM E8 standard [17].

Besides standard mechanical properties under tension (yield stress *σ*_YS_, ultimate stress *σ*_UTS_ and elongation ε), the parameter of relative elongation to failure ε (total elongation) was divided into two components, uniform relative elongation ε_u_ (before necking) and non-uniform one ε_n_ (during necking until fracture, that is, post-necking relative elongation), ε = ε_u_ + ε_n_. Uniform elongation was determined as the elongation at the maximum load.

**Figure 4:**
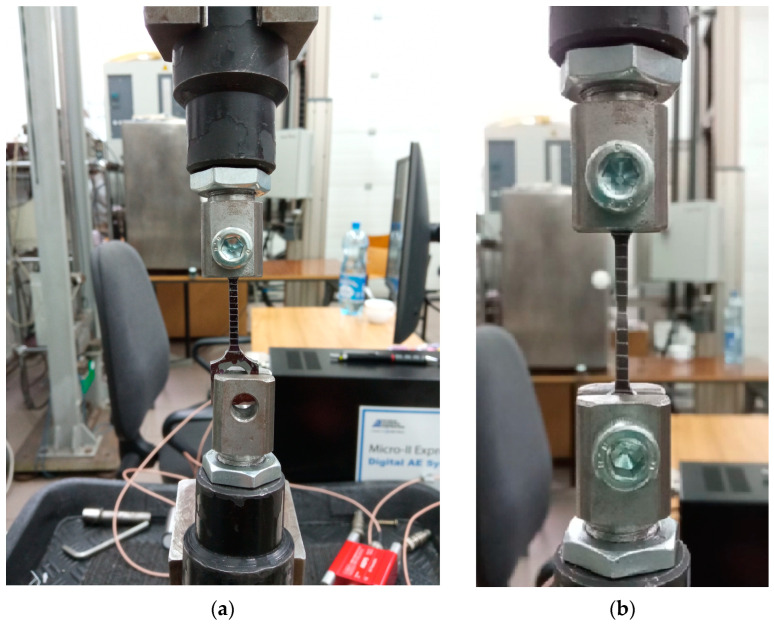
Photographs of the tensile test specimens before test (**a**) and after test (**b**).

Experimental tensile tests were carried out using the Zwick-100 testing machine equipped with an automated control data recording system (Figure 4). Markings were made every 2.0 mm on the measuring section of the specimens. During the tensile test, the elongation on the measuring part was recorded using a video camera. The real-time recording of the elongation made it possible to determine the increment of the measuring section, the uniform elongation—ε_u_ and non-uniform one—ε_n_. Additional markings on the measuring sections, together with the use of video recording, also made it possible to determine the actual strain in the material specimens at each stage of its loading [44].

Fractographic and metallographic testing has been performed using SEM–JEOL JSM-7100F.

## 3. Experimental Results and Discussion

### 3.1. Impact Toughness Testing

The impact strength values of the studied steels from two crane units showed a remarkable difference (Table 3). Thus, impact toughness of steel A was lower by approx. 50% for both longitudinal and transversal specimens compared to that of steel B.

**Figure 5:**
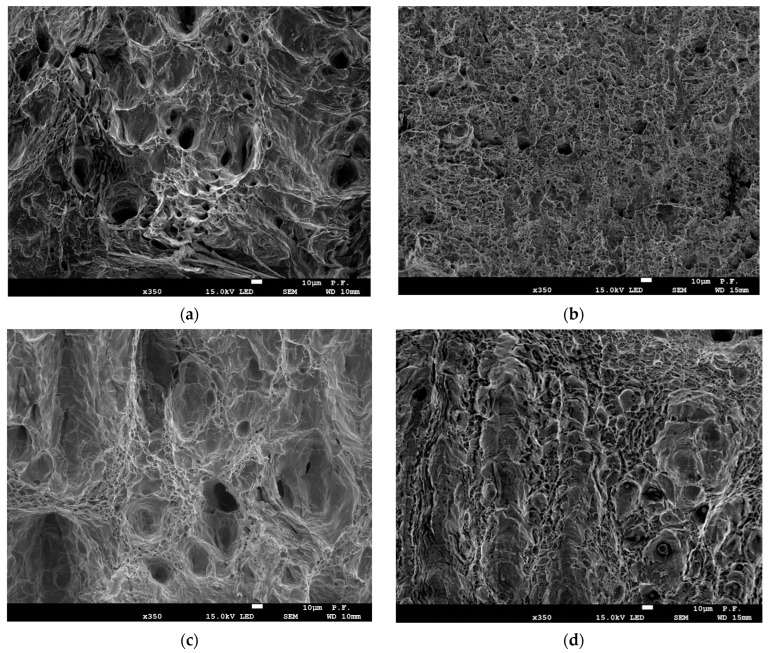
Fracture surfaces of the longitudinal L-T (**a**,**b**) and transverse T-L (**c**,**d**) specimens after impact toughness testing: (**a**,**c**)—steel A; (**b**,**d**)—steel B.

The impact fracture surfaces of the longitudinal and transverse specimens of the studied steels are presented in Figure 5. The investigated specimens showed mostly ductile fracture. However, delaminations were also revealed for both steels, which were more pronounced for steel A, especially for the transverse specimen, compared with steel B (Figure 5c,d). This indicates that steel A operated under higher stresses (Table 1) and, characterized by lower resistance to brittle fracture (Table 3), is susceptible to low-energy delamination along the rolling direction (Figure 5c).

The results confirmed the suggestion about the crucial effect of operational cyclic stresses on the steel state. Indeed, the KCV level of steel A with higher cyclic stresses is twice as low in comparison with steel B (Table 1 and Table 3). In addition, the impact toughness of transverse specimens KCV_T-L_ is approx. 2.5 times less than that of the longitudinal ones KCV_L-T_ for both studied steels. This could be considered as an indicator of the operational degradation of steel since the ratio KCV_T_/KCV_L_ for rolled steel is usually close to 0.7 and rarely drops to 0.5 [25,26,27]. Similar results have been obtained by other researchers [45] and explained by more pronounced delaminations along the texture layers in more degraded steel. However, the present study is not aimed at elucidating the mechanisms of the operational degradation of the tested pieces of metal; further research is focused on its consequences, trying to specify the plasticity parameters suitable for assessing the degree of operational degradation of portal crane steel.

Thus, the chosen material for the investigation reveals signs of operational degradation. It can be suggested that steel A is characterized by a higher degradation degree than steel B.

### 3.2. Stress–Strain Curves

**Figure 6:**
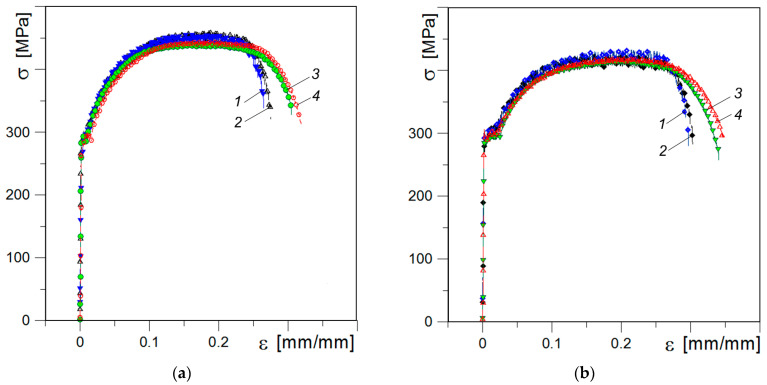
Stress–strain curves of steel A (**a**) and B (**b**) for thinner (*1*, *2*) and thicker (*3*, *4*) specimens of transverse (*1*, *3*) and longitudinal (*2*, *4*) orientation.

Figure 6 depicts the experimentally measured stress-strain curves of the tested specimens. The average values of strength and plasticity characteristics determined by tensile testing of the investigated steels are presented in Table 4.

**Figure 7:**
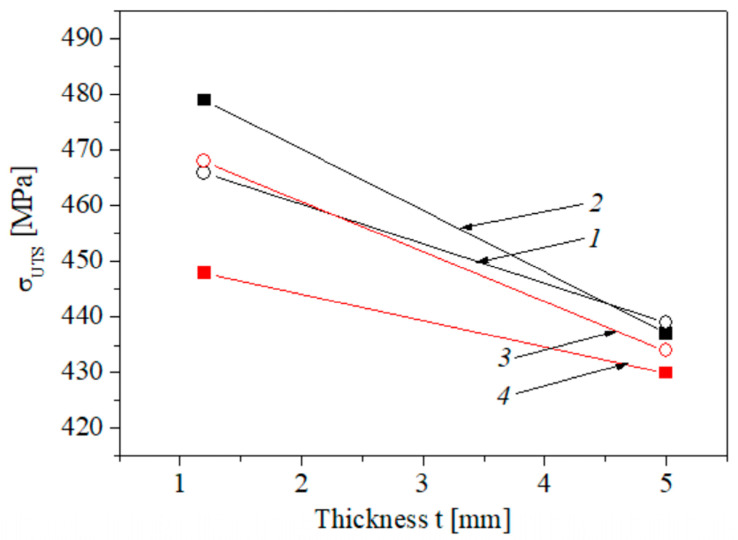
The average strength of steel A (*1*, *2*) and B (*3*, *4*) determined using specimens of transverse (*1*, *3*) and longitudinal (*2*, *4*) orientation.

Ultimate strength divides the stress-strain curve into two parts: the former illustrates uniform elongation and the latter—non-uniform elongation under necking (post-necking elongation). In general, the changes in ultimate strength due to the considered factors (the operational stresses, the cutting direction of specimens and their thickness) are small, but some trends can be noted (Figure 7): ultimate strength characteristics are slightly higher for steel A compared with that determined for steel B. In our case, definitely higher σ_UTS_ values were obtained for thinner specimens, especially for more embrittled material A. With increasing thickness, both the strain-hardening part and the necking portion in the stress-strain curves of the studied steels are prolonged to a higher strain resulting in a larger overall ductility (Figure 6a,b).

As can be seen from Figure 7, steel B showed a pronounced strength anisotropy depending on the plane of fracture propagation relative to the rolling direction of the plate at testing specimens with different thickness. For material A, anisotropy in ultimate strength was only observed when testing the thinner specimens.

The main attention has been paid to the analysis of steel plasticity depending on its operational condition (comparison of materials A and B), texture (longitudinal and transversal specimens) and specimen thickness.

### 3.3. Elongation

**Figure 8:**
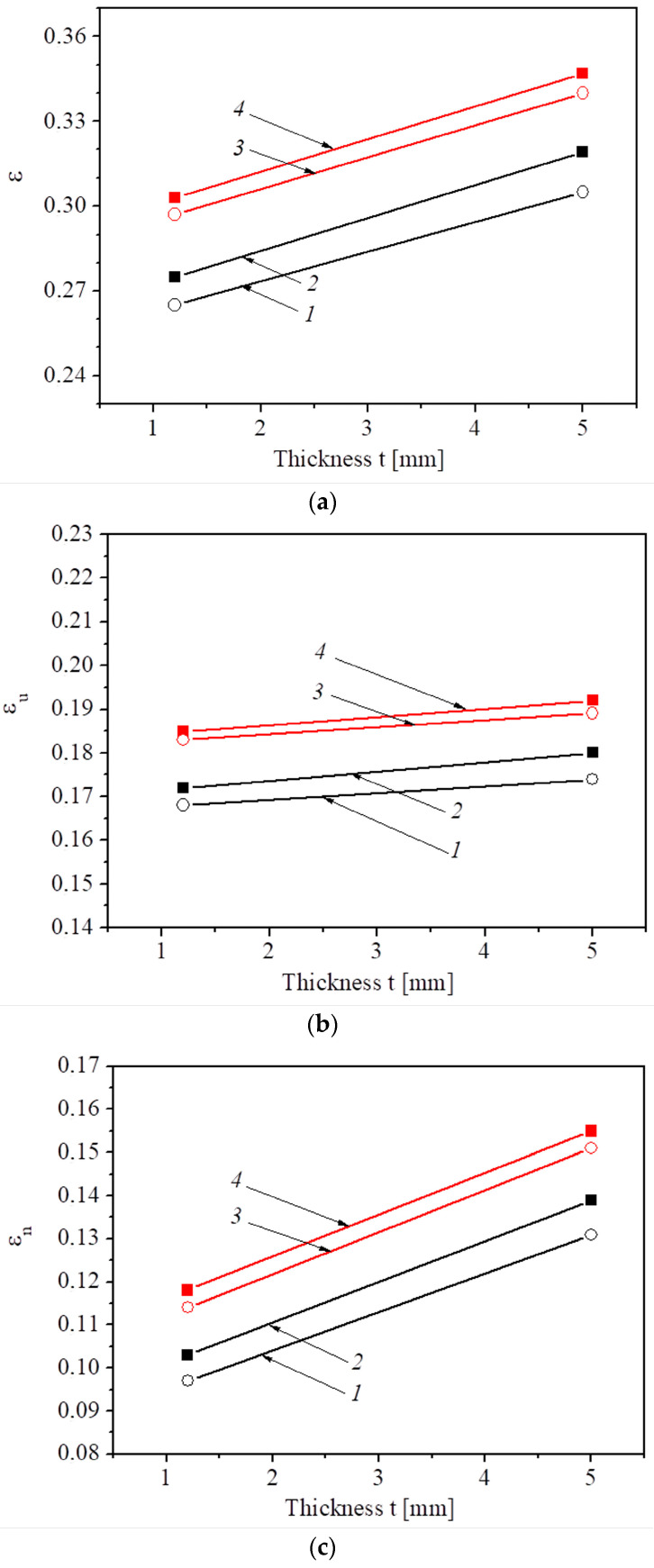
The average total elongation (**a**), uniform elongation (**b**), and non-uniform elongation (**c**) of steel A (*1*, *2*) and B (*3*, *4*) determined using specimens of transverse (*1*, *3*) and longitudinal (*2*, *4*) orientation.

Steel B, with a higher brittle fracture resistance KCV than steel A, expectedly has higher plasticity determined by total elongation ε. This regularity, in general, is valid regardless of the cutting direction of the specimens and their thickness (Figure 8a). Concerning the influence of specimen thickness on their plasticity, the total elongation of both steel states is significantly lower for thinner specimens regardless of their cutting direction due to a larger volume of the material in the gauge section in a thicker specimen, resulting in a higher elongation. Therefore, the specimen with higher thickness better resists crack growth and fracture during necking.

The discussed regularities of the influence of the operational conditions, specimen cutting direction and their thickness on steel plasticity are the results of the influence of these factors on its components ε_u_ and ε_n_, which reflect the features of deformation and fracture of specimens at the stages of uniform elongation and necking. Significant changes in either component inevitably contribute to the parameter of total elongation ε.

Uniform elongation is sensitive to the considered factors with the same regularities as for total elongation ε: the value ε*_u_* is noticeably lower for more embrittled steel A regardless of the specimen thickness (Figure 8b). Obviously, this component plays a key factor in the formation of total elongation ε. Concerning the effect of specimen thickness on the parameter ε*_u_*, the regularity is the same as in the case of the total elongation ε: uniform elongation is somewhat lower for thinner specimens regardless of the steel state and specimen cutting. The obtained results are consistent with the research [7,21], demonstrating the effect of the size factor on the plasticity of the metal at the stage of uniform elongation: it increases with increasing specimen thickness.

The anisotropy of uniform elongation was insignificant for both studied steels regardless of the thickness of specimens (Figure 8b). The results indicate a stronger manifestation of the factors leading to the transition from uniform elongation to necking when testing specimens with a thickness of 1.2 mm. Since the beginning of the neck formation is identified by reaching the maximum stress on the stress-strain curve and associated with the development of micro defects in the specimen cross-section, then the role of these defects in the thin specimen is manifested at a lower deformation.

Analyzing the influence of specimen thickness on non-uniform elongation ε_n_ of specimens cut out from the tested sections of the crane in different orientations (Figure 8c) revealed that this parameter for both steels is significantly higher for thicker specimens regardless of their orientation relative to the rolling direction. This result is consistent with findings of the research [7] demonstrating an increase of both uniform and post-necking elongation with increasing specimen thickness. Due to a low aspect ratio width/thickness in the tested specimens, a diffuse necking mode could be expected [19,46,47].

The comparison of materials A and B (Figure 8c) indicates a high sensitivity of the parameter ε_n_ in the assessment of their operational changes in a metal state regardless of the thickness of the tested specimens. In addition, the effect of texture on the parameter ε_n_ is also clearer: for both tested materials, elongation under necking is lower in the case of transversal specimens; the effect is more significant in more embrittled steel. This means that the assessment of the operational changes in a metal state is the most expedient by the parameter of non-uniform elongation ε_n_ using transversal specimens. Such a conclusion is consistent with the estimation of operational degradation of steel by the resistance to brittle fracture (impact toughness and fracture toughness) [37,42].

The results summarized in Figure 8 made it possible to identify the following regularities. The plasticity characteristics of the steel are more sensitive to the operational changes in metal state, texture, and specimen thickness than its strength but less sensitive than impact toughness (the effect of specimen thickness was not considered when evaluating KCV) which varied from 60 J/cm^2^ to 310 J/cm^2^ (by more than 5 times) whereas the elongation parameters changed by an approx. 10–25%.

### 3.4. Fractographic Analysis of Tensile Failures

**Figure 9:**
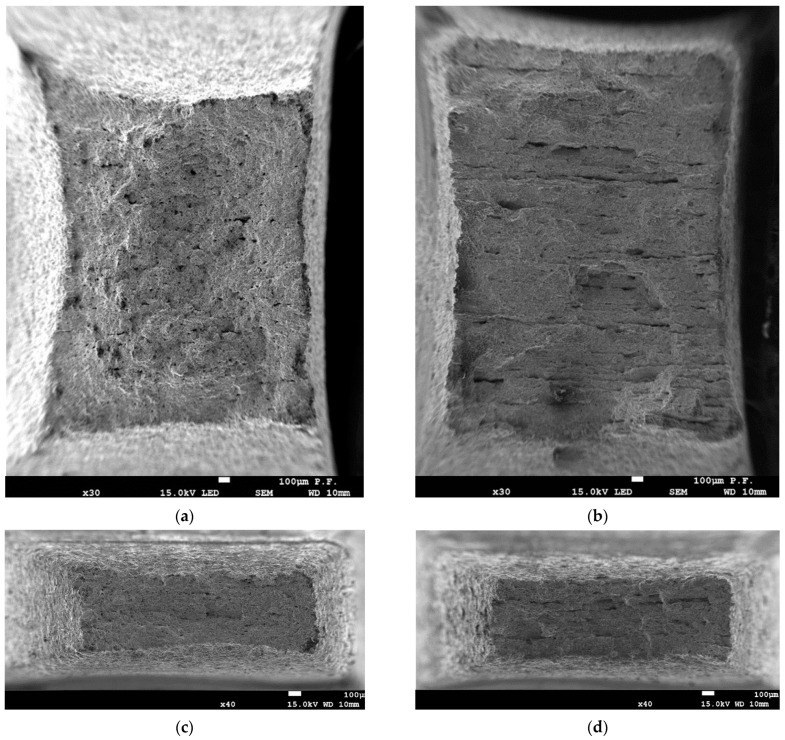
Examples of the macro fracture surfaces of specimens made from steel A with a thickness of 5.0 mm (**a**,**b**) and 1.2 mm (**c**,**d**), cut out along (**a**,**c**) and across (**b**,**d**) the rolling direction.

The regularities of plastic deformation of specimens have been analyzed taking into account their macro and micro fractographic features. Figure 9 illustrates typical macro fracture surfaces of tensile specimens of different thicknesses and cutting directions on the example of more embrittled steel A, providing a general view of the cup and cone fracture. The component “cup” (central part of the fracture surface) is characterized by delamination, which is more pronounced for the transversal specimens (Figure 9b,d). It indicates that delaminations formed either during operation or mechanical testing directly contribute to the formation of the fracture surface. Concerning the size of the component “cone” (on the sides), it is visually smaller for the transversal specimens.

Even a cursory analysis of the ratio between “cup” and “cone” component sizes revealed a crucial distinction for the specimens of different thicknesses (Figure 9). This is essential since “cup” is formed by tearing under triaxial stress conditions, whereas “cone” is by the shear mechanism under the plane stress state. It can be noted that the total size of “cone” is approx. 1/3 of the size of “cup” for thicker specimens (~1.2 mm), whereas, for thinner ones, this ratio reaches 1/2 (~0.6 mm). However, the question about what is crucial in the steel plasticity, the total size of “cone” or its relative value (concerning the “cup” size), should be further clarified.

**Figure 10:**
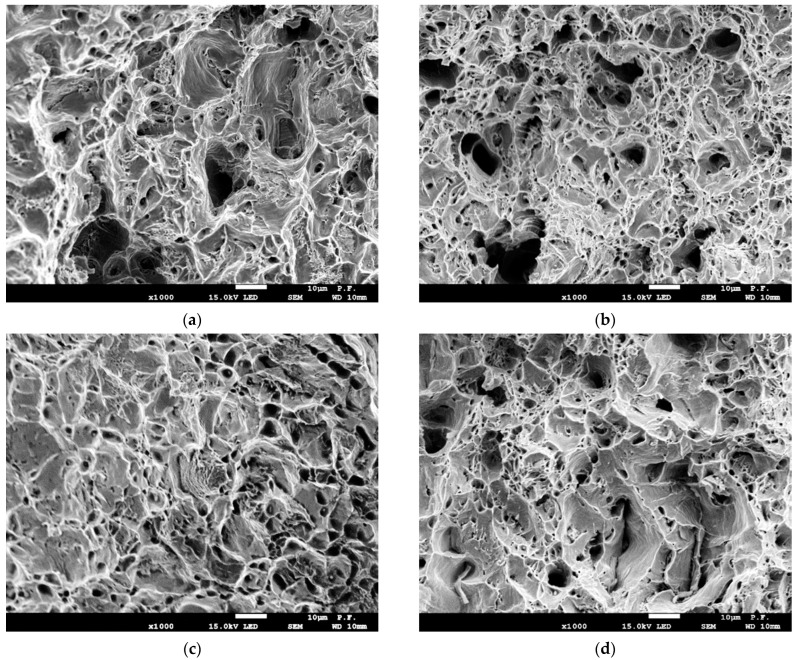
Fracture surfaces of specimens with a thickness of 5.0 mm from steel A (**a**,**c**) and steel B (**b**,**d**), cut out along (**a**,**b**) and across (**c**,**d**) the rolling direction.

Micro fractographic analysis has been performed for specimens of both steel states cut out longitudinally and transversely relative to the rolling direction. A typical fracture mechanism for all analyzed specimens is micro void coalescence (Figure 10). Deep small voids prevail on the fracture surface of the longitudinal specimen of steel B. They were formed by stretching with subsequent fracture of partitions between adjacent pores near carbides and inclusions (Figure 10b). In contrast, the fracture surface of more embrittled steel A contains mainly shallow and larger voids formed by the shear mechanism (Figure 10a). In addition, the amount of roundish fracture elements (delaminations), initiated from a chain of small non-metallic inclusions and formed by shear, is bigger for the steel A, and they are larger (30–40 μm in diameter). This facilitated the localization of deformation, resulting, thus, in less elongation for more embrittled steel A by approx. 8%.

The amount of delaminations along the chains of non-metallic inclusions is significantly bigger (Figure 10d) for the transversal specimen of steel B compared to the longitudinal one although the fracture of partitions between adjacent delaminations is ductile with the formation of equiaxed voids and clearly outlined tearing edges. Concerning the transversal specimen of more embrittled steel A, the role of shear in the fracture of partitions between delaminations is more pronounced (Figure 10c) despite the predominance of micro-void relief. As a result, the height difference in the fracture surface relief is lower, and tearing edges were formed at the transitions between adjacent areas of micro shear. Thus, the operational changes in the state of steel A are more pronounced on the fracture surface of the transversal specimen, and they manifest by enhancing the role of shear in the fracture. Since the shear is the final stage in the fracture process of flat tensile specimens, then its predominance on the fracture surface of the transversal specimen of steel A is in agreement with its lower plasticity compared to steel B by approx. 10%.

### 3.5. Future Research Prospects

Further research will be focused on the influence of the shape and dimensions of specimens loaded by uniaxial tension on the plasticity characteristics of the material under non-uniform strain. Experimental studies will be supplemented with numerical modelling to clarify the influence of the specimen shape on the evolution of stress and strain fields occurring during the neck formation. As a result, formulas will be obtained which describe the relationship between true stresses and strains, taking into account the specific dimensions of structural elements. The research will enable determining the limitations and proper solutions for the correct assessment of the operational changes in a metal state for the rolled structural steels when the structure is made of sheets of the same steel grade but with different thicknesses, taking into account the anisotropy of its mechanical properties.

## 4. Conclusions

Based on the investigations of long-term-operated sheet steel of a portal crane, the influence of certain factors, i.e., the operational conditions, specimen cutting direction and thickness, on strength and plasticity has been analyzed, distinguishing the components of uniform and non-uniform elongation. The performed analysis made it possible to formulate the following conclusions.

Strength indicators are not sensitive to the considered factors (the operational stresses, the cutting direction of specimens and their thickness). However, a clear trend of higher ultimate strength for thinner specimens is noticed, especially in the case of more brittle material.

Plasticity of the tested steel specimens, in general, is sensitive to the influence of the above-mentioned factors but less sensitive than impact toughness KCV. Uniform elongation is slightly lower for thinner specimens regardless of the state of the investigated steel and the orientation of specimens relative to the rolling direction. Among the plasticity parameters, non-uniform elongation is the most effective for assessing the operational changes in the steel regardless of thickness of specimens. In addition, post-necking elongation is lower in the case of transversal specimens, and the effect is more significant in more embrittled steel.

The results demonstrate that it would be advantageous to use the parameter of the non-uniform elongation ε_n_ determined using transversal specimens as the most sensitive for the assessment of the operational changes in a metal state for rolled sheet steels among the tensile properties.

## Figures and Tables

**Table 1:** Cyclic stress ranges at the surface of considered crane units.

Steel	Crane Unit	Sheet Thickness [mm]	Cyclic Stress Range Δσ*_e_* [MPa]
A	Jib	16.0	130.0
B	Boom	12.0	55.0

**Table 2:** Specimen marking depending on their cutting orientation and thickness.

Steel	Specimen Orientation	Specimen Code for t_1_ = 5.0 mm	Specimen Code for t_2_ = 1.2 mm
A	Longitudinal	A-L-t_1_	A-L-t_2_
	Transverse	A-T-t_1_	A-T-t_2_
B	Longitudinal	B-L-t_1_	B-L-t_2_
	Transverse	B-T-t_2_	B-T-t_2_

**Table 3:** Impact toughness experimentally observed for studied steels.

Steel	Crane Unit	KCV_L-T_ [J/cm^2^] ^1^	KCV_T-L_ [J/cm^2^] ^2^
A	Jib	159	60
B	Boom	310	123

^1^ KCV_L-T_—impact toughness determined using longitudinal specimens. ^2^ KCV_T-L_—impact toughness determined using transverse specimens.

**Table 4:** Mechanical properties of the tested specimens evaluated by tensile testing.

Specimen Code	σ_YS_ [MPa]	*σ*_UTS_ [MPa]	ε [mm/mm]	ε_u_ [mm/mm]	ε_n_ [mm/mm]
A-L-t_1_	282	437	0.319	0.180	0.139
A-T-t_1_	283	439	0.305	0.174	0.131
B-L-t_1_	298	430	0.347	0.192	0.155
B-T-t_1_	300	434	0.340	0.189	0.151
A-L-t_2_	297	479	0.275	0.172	0.103
A-T-t_2_	300	466	0.265	0.168	0.097
B-L-t_2_	305	448	0.303	0.185	0.118
B-T-t_2_	322	468	0.297	0.183	0.114

## Data Availability

Data available on the request to the corresponding author.

## References

[1] Kumar N., Mishra R.S., Huskamp C.S., Sankaran K.K. (2011). Critical grain size for change in deformation behavior in ultrafine grained Al–Mg–Sc alloy. Scripta Mater..

[2] Zvirko O.I., Tsyrulnyk O.T., Dzioba I., Kret N.V., Lipiec S. (2021). Influence of the structural features of steels of casing pipes on their mechanical properties and hydrogen brittleness. Mater. Sci..

[3] Zvirko O., Tsyrulnyk O., Lipiec S., Dzioba I. (2021). Evaluation of corrosion, mechanical properties and hydrogen embrittlement of casing pipe steels with different microstructure. Materials.

[4] Morris J.W. (2001). The Influence of Grain Size on the Mechanical Properties of Steel. https://escholarship.org/uc/item/88g8n6f8.

[5] Morquio A., Riera J.D. (2004). Size and strain rate effects in steel structures. Eng. Struct..

[6] Motra H.B., Hildebrand J., Dimmig-Osburg A. (2014). Assessment of strain measurement techniques to characterise mechanical properties of structural steel. Eng. Sci. Technol. Int. J..

[7] Zhao Y.H., Guo Y.Z., Wei Q., Topping T.D., Dangelewicz A.M., Zhu Y.T., Langdon T.G., Lavernia E.J. (2009). Influence of specimen dimensions and strain measurement methods on tensile stress–strain curves. Mater. Sci. Eng. A.

[8] Yuan W.J., Zhang Z.L., Su Y.J., Qiao L.J., Chu W.Y. (2012). Influence of specimen thickness with rectangular cross-section on the tensile properties of structural steels. Mater. Sci. Eng. A.

[9] Motra H.B., Hildebrand J., Dimmig-Osburg A. (2014). Influence of specimen dimensions and orientation on the tensile properties of structural steel. Mater. Test..

[10] Kumar K., Pooleery A., Madhusoodanan K., Singh R.N., Chatterjee A., Dutta B.K., Sinha R.K. (2016). Optimisation of thickness of miniature tensile specimens for evaluation of mechanical properties. Mater. Sci. Eng. A.

[11] Trieu K., Wang X., Zhang X. (2019). The effect of size on the mechanical properties of rolled specimen: Predictions and experimental evaluation. Mater. Res. Express.

[12] Zhang L., Harrison W., Yar M.A., Brown S.G.R., Lavery N.P. (2021). The development of miniature tensile specimens with non-standard aspect and slimness ratios for rapid alloy prototyping processes. J. Mater. Res. Technol..

[13] Kazakeviciute J., Rouse J.P., De Focatiis D.S.A., Hyde C.J. (2022). Small specimen techniques for estimation of tensile, fatigue, fracture and crack propagation material model parameters. J. Strain Anal. Eng. Des..

[14] Neuenschwander M., Knobloch M., Fontana M. (2017). Elevated temperature mechanical properties of solid section structural steel. Constr. Build. Mater..

[15] Dzioba I., Lipiec S. (2019). Fracture mechanisms of S355 steel—Experimental research, FEM simulation and SEM observation. Materials.

[16] Tu S., Ren X., He J., Zhang Z. (2019). Experimental measurement of temperature-dependent equivalent stress-strain curves of a 420 MPa structural steel with axisymmetric notched tensile specimens. Eng. Fail. Anal..

[17] (2016). Standard Test Methods for Tension Testing of Metallic Materials.

[18] (2019). Metallic Materials—Tensile Testing—Part 1: Method of Test at Room Temperature.

[19] Goh T.N., Shang H.M. (1982). Effects of shape and size of tensile specimens on the stress-strain relationship of sheet-metal. J. Mech. Work. Technol..

[20] Takeda Y., Kiattisaksri C., Aramaki M., Munetoh S., Furukimi O. (2017). Effects of specimen thickness in tensile tests on elongation and deformation energy for industrially pure iron. ISIJ Int..

[21] Suh C.H., Yun-Chul J., Kim Y.S. (2010). Effects of thickness and surface roughness on mechanical properties of aluminum sheets. J. Mech. Sci. Technol..

[22] Nizhnik S.B. (1997). Effect of structure and texture on the anisotropy of strength properties arising in the rolling of steel tubes. Strength Mater..

[23] Joo M.S., Suh D.W., Bhadeshia H.K.D.H. (2013). Mechanical anisotropy in steels for pipelines. ISIJ Int..

[24] Masoumi M., Herculano L.F.G., de Abreu H.F.G. (2015). Study of texture and microstructure evaluation of steel API 5L X70 under various thermomechanical cycles. Mater. Sci. Eng. A.

[25] Hutchinson B. (2015). Critical assessment 16: Anisotropy in metals. Mater. Sci. Technol..

[26] Nykyforchyn H., Zvirko O., Tsyrulnyk O., Kret N. (2017). Analysis and mechanical properties characterization of operated gas main elbow with hydrogen assisted large-scale delamination. Eng. Fail. Anal..

[27] Zvirko O.I., Kret N.V., Tsyrulnyk O.T., Vengrynyuk T.P. (2018). Influence of textures of pipeline steels after operation on their brittle fracture resistance. Mater. Sci..

[28] Beltran-Zuñiga M.A., González-Velázquez J.L., Rivas-López D.I., Dorantes Rosales H.J., Hernández-Santiago F. (2018). Effect of microstructure and crystallographic texture on the toughness anisotropy of API 5L X46 steel. Fatigue Fract. Eng. Mater. Struct..

[29] Tankoua F., Crépin J., Thibaux P., Cooreman S., Gourgues-Lorenzon A.-F. (2018). Quantification and microstructural origin of the anisotropic nature of the sensitivity to brittle cleavage fracture propagation for hot-rolled pipeline steels. Int. J. Fract..

[30] Das Bakshi S., Javed N., Sasidhar K.N., Dhande T., Sharma V., Mukherjee M. (2018). Effect of microstructure and crystallographic texture on mechanical anisotropy of Ti-Nb microalloyed hot rolled 800MPa HSLA steel. Mater. Charact..

[31] Marushchak P.O., Kret N.V., Bishchak R.T., Kurnat I.M. (2019). Influence of texture and hydrogenation on the mechanical properties and character of fracture of pipe steel. Mater. Sci..

[32] Lesiuk G., Rymsza B., Rabiega J., Correia J.A.F.O., De Jesus A.M.P., Calcada R. (2019). Influence of loading direction on the static and fatigue fracture properties of the long term operated metallic materials. Eng. Fail. Anal..

[33] Nemchuk O.O., Nesterov O.A. (2020). In-service brittle-fracture resistance degradation of steel in a ship-to-shore gantry crane. Strength Mater..

[34] Semenov P.O., Pustovyi V.M. (2020). Complex diagnostics of the state of operated elements of a grab reloader. Mater. Sci..

[35] Zvirko O.I. (2021). In-service degradation of structural steels (A survey). Mater. Sci..

[36] Dzioba I., Zvirko O., Lipiec S., Bolzon G., Gabetta G., Nykyforchyn H. (2021). Assessment of operational degradation of pipeline steel based on true stress–strain diagrams. Degradation Assessment and Failure Prevention of Pipeline Systems. Lecture Notes in Civil Engineering.

[37] Nykyforchyn H., Zvirko O., Dzioba I., Krechkovska H., Hredil M., Tsyrulnyk O., Student O., Lipiec S., Pala R. (2021). Assessment of operational degradation of pipeline steels. Materials.

[38] Pustovyi V.M., Semenov P.O., Nemchuk O.O., Hredil M.I., Nesterov O.A., Strelbitskyi V.V. (2022). Degradation of steels of the reloading equipment operating beyond its designed service life. Mater. Sci..

[39] Nykyforchyn H., Pustovyi V., Zvirko O., Semenov P., Hredil M., Nemchuk O., Oliynyk O., Tsyrulnyk O. (2022). Analysis of operational factors affecting the serviceability of seaport hoisting and transporting equipment. Procedia Struct. Integr..

[40] Zvirko O., Mytsyk B., Nykyforchyn H., Tsyrulnyk O., Kost’ Y. (2022). Application of the various methods for assessment of in-service degradation of pipeline steel. Mech. Adv. Mater. Struct..

[41] Nykyforchyn H., Tsyrulnyk O., Zvirko O., Hredil M. (2020). Role of hydrogen in operational degradation of pipeline steel. Procedia Struct. Integr..

[42] Nykyforchyn H., Zvirko O., Hredil M., Krechkovska H., Tsyrulnyk O., Student O., Unigovskyi L. (2022). Methodology of hydrogen embrittlement study of long-term operated natural gas distribution pipeline steels caused by hydrogen transport. Frat. Integrità Strutt..

[43] (2018). Standard Test Methods for Notched Bar Impact Testing of Metallic Materials.

[44] Dzioba I., Lipiec S., Pala R., Furmanczyk P. (2021). On characteristics of ferritic steel determined during the uniaxial tensile test. Materials.

[45] Nemchuk O.O., Krechkovska H.V. (2019). Fractographic substantiation of the loss of resistance to brittle fracture of steel after operation in the marine gantry crane elements. Metallofiz. Noveishie Tekhnol..

[46] Khan I.A., Srivastava A., Needleman A., Benzerg A.A. (2021). An analysis of deformation and failure in rectangular tensile bars accounting for void shape changes. Int. J. Fract..

[47] Tu S., Ren X., He J., Zhang Z. (2020). Stress–strain curves of metallic materials and post-necking strain hardening characterization: A review. Fatigue Fract. Eng. Mater. Struct..

